# Determination of geographical origin Turkish hazelnuts according to fatty acid composition

**DOI:** 10.1002/fsn3.595

**Published:** 2018-02-02

**Authors:** Fikret Tüfekci, Şükrü Karataş

**Affiliations:** ^1^ Department of Food Engineering Istanbul Aydin University Istanbul Turkey

**Keywords:** Black Sea, fatty acid composition, geographical origin, hazelnut (*Corylus avellena* L.), linear discriminant analysis

## Abstract

This study focuses on detecting geographical origin of round type hazelnut (*Corylus avellena* L.) in Turkey using fatty acid (FA) composition. The samples were collected from Western, Central, and Eastern Black Sea regions between 2015 and 2016. FA profiles were determined by gas chromatography (GC), and most abundant fatty acid was oleic acid (C18:1) followed by linoleic acid (C18:2), palmitic acid (C16:0), and stearic acid (C18:1). The effect of geographical origin on the fatty acid profile of hazelnut oils was statistically analyzed by one‐way ANOVA and linear discriminant analysis (LDA). The results showed that the Central Black Sea region had high content of total saturated fatty acids (%8.45), total monounsaturated fatty acids (%83.54), low content of total polyunsaturated fatty acids (%7.85), and Eastern Black Sea region had high content of linoleic (%9.10) and linolenic acid (%0.096). Six fatty acids (C16:1, C18:1, C18:2, C18:3, C20:0, and C20:1) identified by LDA provide 86.2% of correct predictions.

## 1 INTRODUCTION

Hazelnut is a good source of oil (50–73%) and rich in unsaturated fatty acids (oleic, linoleic, linolenic acid etc.) (Köksal, Artik, Şimşek, & Güneş, 2006). It has been reported that a dietary culture rich in monounsaturated fat content (such as oleic acid) (i) increases the amount of high‐density lipoproteins (HDL) while reducing the amount of low‐density lipoproteins (LDL) and (ii) helps prevent cholesterol‐induced cardiovascular disease (Oliveira et al., 2008). Orsavova, Misurcova, Ambrozova, Vicha, and Mlcek (2015) reported that oleic acid enhances insulin resistance and anti‐inflammatory effect as opposed to polyunsaturated fatty acids. Hazelnut is also an important source of nutrients for the human diet with high energy, protein, Vitamin E, and natural phytosterols (Parcerisa, Richardson, Rafecas, Codony, & Boatella, 1998).

Turkey is the world largest hazelnut‐producing country, contributing 63% of the total production (750,000 tons in 2014) followed by Italy (12%), Georgia (4.8%), USA (4.5%), and Azerbaijan (3.8%) (FAOSTAT, 2016). Hazelnut farming in Turkey is carried out in almost all of the Black Sea region and a part of the Marmara region (cities with a coast to the Black Sea; Sakarya and Kocaeli). In Turkey, 17 varieties of cultivars and a wild nut, known as “raw hazelnut,” are cultivated (Köksal et al., 2006). The Turkish hazelnut contains three groups: (1) round (i.e., Kargalak, Çakıldak, Palaz, Tombul, Foşa, Kalınkara, Uzunmusa, Mincane, Cavcava, Kan); (2) sharp (i.e., İncekara, Acı, Kuş); and (3) almond (i.e., Yuvarlak Badem, Yassı Badem, Değirmendere) (Black Sea Hazelnut Exporter's Union, 2012).

The aim of this study was to predict the geographical origin of Turkish hazelnuts (*Corylus avellena* L.) which are cultivated in Black Sea region to correspond the fatty acid composition by statistically analyzing using one‐way ANOVA and linear discrimination analysis (LDA) and to estimate diversity of the hazelnut related to with fatty acid composition and to compare this properties to appropriate other countries.

## 2 EXPERIMENTAL

### 2.1 Hazelnut samples

The hazelnut samples were randomly selected from local hazelnut traders. The samples were harvested in September of 2015 and 2016, and the latitude of the collection orchard is 41° 07′ 07″ N, and 39° 92′ 27″ E., which represents the Eastern, Western, and Central Black Sea regions of Turkey**.** Details about origin of hazelnut including regions are given in Table 1. At least six hazelnut bags from each region and a total of 87 samples were collected into plastic bags of one kg which were kept at −20°C to prevent quality loses until analysis.

**Table 1:** Origin of hazelnut samples

Origin	Number of samples	
	2015	2016
Eastern Black Sea		
Trabzon	6	6
Giresun	11	10
Central Black Sea		
Ordu	6	7
Samsun	8	6
Western Black Sea		
Düzce	3	3
Zonguldak	4	3
Sakarya	8	6

### 2.2 Oil extraction

Fifty grams of each sample of hazelnut was manually cracked and shelled, and kernels were grinded finely using a coffee grinder (Waring America, model HGTWTS3). The crude oil was extracted with petroleum ether (bp 40–60°C) using Soxhelet extractor at 55°C for 15 h, and the remnant solvent was removed with a vacuum rotary evaporator (Heidolph Laborota 4010, Germany) at 40°C about 15 min (ISO 661:^2003^(E), 2003).

### 2.3 Sample preparation

The FAMEs were prepared by vigorously shaking a solution in n‐heptane (0.1 g in 7 ml) with 0.75 ml of 2N methanolic potassium hydroxide (Merck, Darmstadt, Germany) and centrifuged (Eppendorf 5804 R, Germany) 3075 x g for 5 min. The 1 ml supernatants were taken into a sample vial for the analysis (ISO 12966–2:^2011^, 2011).

### 2.4 Gas chromatography

The analysis was performed at Agilent 6890N gas chromatography system (GC) (Agilent, Waldbronn, Germany) equipped with flame ionization detector (FID) and JB Scientific DB‐23 column (60 m × 0.25 mm × 0.25 μm) (ISO 12966–2:^2011^, 2011). Temperature of front inlet and detector were adjusted to 250°C and 260°C, and the flow rate of the carrier gas (nitrogen) was set at 1.4 ml/min.

Injection volume was 0.2 μl with a split ratio of 1:40. The column temperature program was performed in four stages. At the first stage, it was held at 130°C for 1 min. At the second stage, the column temperature was increased to 170°C with a rate of 6.5°C/min. At the third stage, the temperature was increased to 215°C with a rate of 2.75°C/min. At the fourth stage, the temperature was held isothermally for 15 min, and finally increased to 230°C with a rate of 15°C/min and held isothermal for 10 min.

**Figure 1:**
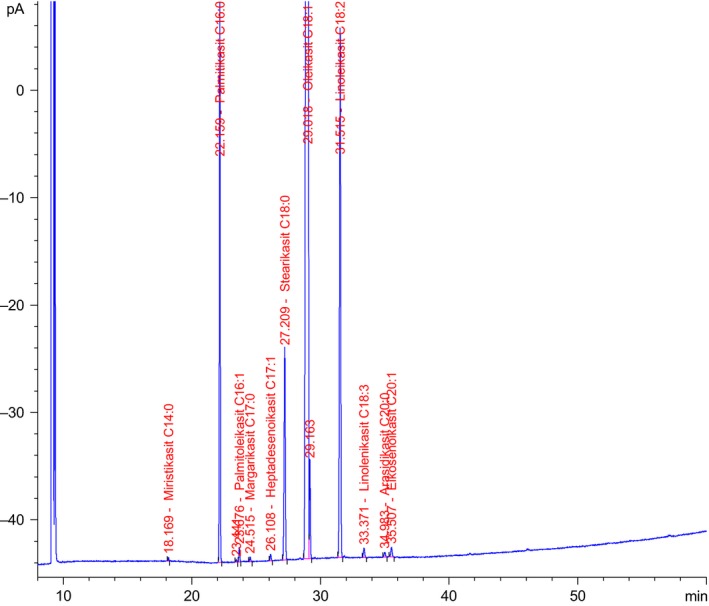
A chromatogram of fatty acid methyl esters an Eastern Black Sea hazelnut sample

The reference standard mixture (Supelco F.A.M.E. 37 Mix, lot: LC‐07964) was analyzed for determining retention times. Analysis was performed in duplicate, and relative amount of each fatty acid was calculated over the total fatty acid content. A typical sample chromatogram is shown in Figure 1.

### 2.5 Statistical analysis

One‐way ANOVA and linear discriminant analysis (LDA) were performed using a statistical package program (SPSS ver. 17.0, 2008; International Business Machines Corp., New Orchard Road, Armonk, New York) for *p *<* *0.05 significance level.

## 3 RESULTS AND DISCUSSIONS

The predominant fatty acid was oleic acid ranging between 82.35% and 83.14%, followed by linoleic acid ranging from 7.75% to 9.10%, palmitic acid 5.18% to 5.65%, and stearic acid from 2.44% to 2.59% as shown in Table 2. Myristic acid, margaric acid, and heptadecanoic acid were present in all samples in trace quantities but not reported. The region of the Central Black Sea had highest oleic acid (83.14%) and lowest linoleic acid values (7.75%) competed to Western and Eastern Black Sea regions.

**Table 2:** Fatty acid profiles (%) of hazelnut (*Corylus avellena L*.) samples from different regions in Turkey

Fatty acids	Eastern Black Sea		Central Black Sea		Western Black Sea	
	Mean	*SE*	Mean	*SE*	Mean	*SE*
C16:0	5.18^b^	0.07	5.65^a^	0.09	5.37^b^	0.09
C16:1	0.157^b^	0.007	0.203^a^	0.007	0.187^a^	0.007
C18:0	2.56^a^	0.05	2.59^a^	0.06	2.44^a^	0.06
C18:1	82.35^a^	0.26	83.14^a^	0.29	82.93^a^	0.29
C18:2	9.10^a^	0.29	7.75^b^	0.32	8.39^ab^	0.32
C18:3	0.096^a^	0.003	0.089^a^	0.003	0.075^b^	0.003
C20:0	0.119^b^	0.002	0.129^a^	0.002	0.118^b^	0.002
C20:1	0.157^a^	0.003	0.154^a^	0.003	0.161^a^	0.003
Total SFAs	7.94^b^	0.11	8.45^a^	0.12	8.01^b^	0.12
Total MUFAs	82.71^a^	0.26	83.54^a^	0.29	83.32^a^	0.29
Total PUFA s	9.19^a^	0.29	7.85^b^	0.32	8.49^ab^	0.32

Each value is a mean of 2 years with standard error (*SE*). Different small letters (a‐b) in a same row indicate significant differences (*p* < .05).

Taş and Gökmen (2015) has been reported that Giresun (which is part of Eastern Black Sea) round hazelnuts had 80.1% oleic acid, 10.91% linoleic acid, 5.7% palmitic acid, and 2.4% stearic acid, respectively. Moreover, Bacchetta et al. (2013) examined that major fatty acid of European hazelnuts was oleic acid (80.63%) followed by 10.57% of linoleic acid, 5.95% of palmitic acid, and 2.48% of stearic acid. These findings were slightly in agreement with our results, and the differences may be due to harvesting season, growing conditions, and locations. On the other hand, Parcerisa et al. (1998) reported that fatty acid profiles of American hazelnut varieties were oleic acid ranged from 77.08% to 80.76%, linoleic acid ranged from 10.46% to 15.55%, palmitic acid ranged from 4.72% to 5.77%, and stearic acid ranged from 1.38% to 3.34%. Rezaei, Bakhshi, Ghazvini, Majd, and Pourghayoumi (2014) reported Iranian hazelnut fatty acid profiles were followed: oleic acid 71.02%, linoleic acid 14.45%, palmitic acid 3.73%, and stearic acid 4.02%, and Derewiaka, Szwed, and Wolosiak (2014) reported a hazelnut species grown in Georgia, oleic acid 77.4%, linoleic acid 8.4%, palmitic acid 10.1%, and stearic acid 3.72%. These data were disagreement with Turkish round type hazelnut features, and these differences may be due to the latitude, variety, environmental conditions, and geographical origin. Alasalvar et al. (2003) stated that the geographical origin, location, species, climate, cultural practices, fertilization, and harvest season have major impact on the fatty acid composition. Furthermore, Pritchard, Eagles, Norton, Salisbury, and Nicolas (2000) reported that when the amount of oleic acid increased in oily seed plants, the amount of linoleic acid and linolenic acid decreased and similarly when the high air temperatures and spring precipitation raised the amount of oleic acid increased. Parcerisa et al. (1993) also reported that the fruit chemical composition is strongly influenced by environmental and growing conditions.

Total monounsaturated fatty acids (MUFAs) are the major group of hazelnut oil due to oleic acid, while total polyunsaturated fatty acids (PUFAs) and total saturated fatty acids (SFAs) were in lower amounts. The MUFAs ranged from 82.71% to 83.54%, PUFAs ranged from 7.85% to 9.19%, and SFAs ranged from 7.94% to 8.45% over all three regions. Another point worth mentioning is that the Central Black Sea region hazelnuts had higher MUFAs (83.54%) and SFAs (8.45%) content. On the other hand, Eastern Black Sea region hazelnuts had lowest SFAs (7.94%) and MUFAs (82.71%) content.

Oliveira et al. (2008) were given total SFAs, MUFAs, and PUFAs content of three hazelnut varieties from Portugal range between 6.97% and 7.77%, 81.12% and 83.0%, 9.99% and 10.63%, respectively. Zytkiewicz et al. (2015) investigated that total SFAs, MUFAs, and PUFAs content of two hazelnut varieties from Poland range from 5.99% to 6.95%, 80.65% to 81.80%, and 11.25% to 13.36%, respectively. These findings were agreement with our MUFAs values but disagreement with our SFA and PUFA values.

Parcerisa et al. (1998) reported total SFAs, MUFAs, and PUFAs content of seven varieties of American hazelnuts range from 6.87% to 8.68%, 77.47% to 81.10%, and 10.64% to 15.66%, respectively. These findings were disagreement with our SFAs, MUFAs, and PUFAs values as well. These differences may be due to geographical origin, climate, varieties, fertilizing, and environmental conditions. Cristofori, Ferramondo, Bertazza, and Bignami (2008) reported that the oil of the fruits grown in cold regions contains more unsaturated fatty acids than the fat of the fruits grown in hot and dry regions.

Discriminant analysis performed discrimination among the three regions of Black Sea in Turkey. Discriminant function 1 (explaining 69.3% of the variability) was positively significantly related to palmitoleic acid, linolenic acid, and arachidic acid and negatively related to oleic acid, linoleic acid, and eicosenoic acid. Discriminant function 2 (explaining 30.7% of the variability) was negatively related to oleic acid, linoleic acid, and arachidic acid and positively related to palmitoleic acid, linolenic acid, and eicosenoic acid (see Table 3).

**Table 3:** Linear discriminant analysis of fatty acid statistical results

Discriminant functions	Eigenvalue	%Variance	Canonical correlation	*p‐*Value
1	1.809	69.3	0.803	0.000
2	0.802	30.7	0.667	0.000

The percentages of predictive probability in the differentiation of origin concerning hazelnut samples for three regions are shown in Table 4. Overall, 86.2% of the cross‐validated cases out of 87 and for the all the regions were indicated and classified correctly: about 93.9% of the Eastern, 70.4% of the Central, and 92.6% for the Western Black Sea. The classification of samples was good especially among the Eastern‐Western and Western‐Central Black Sea: 75 cases of 87 were identified correctly and 12 cases were incorrectly classified.

**Table 4:** Linear discriminant analysis of fatty acid classification of results

		Region	Predicted Group Origin			Total
			Eastern Black Sea	Central Black Sea	Western Black Sea	
Original	Count	East Black Sea	31	1	1	33
		Middle Black Sea	7	19	1	27
		West Black Sea	1	1	25	27
	%	East Black Sea	93.9	3.0	3.0	100.0
		Middle Black Sea	25.9	70.4	3.7	100.0
		West Black Sea	3.7	3.7	92.6	100.0

86.2% of original grouped cases correctly classified.

**Figure 2:**
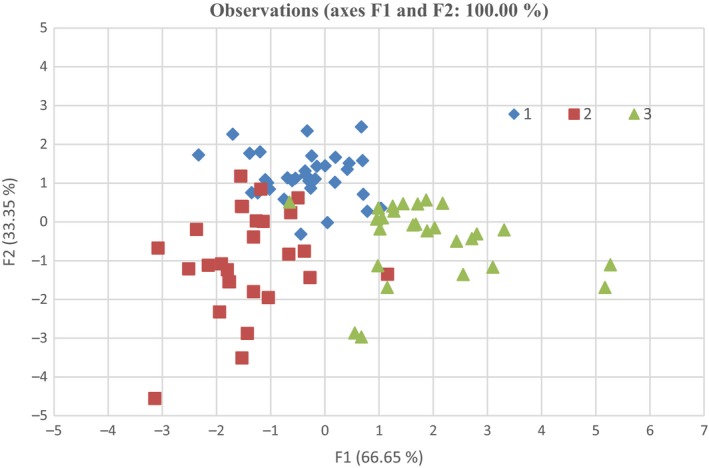
Plot of the linear discriminant analysis of hazelnut samples (1: Eastern 2: Central, and 3; Western Black Sea regions)

Almost whole Western Black Sea region samples can be differentiated from Eastern Black Sea and Central Black Sea region samples (see Figure 2). The high misclassification rate in the Central Black Sea region might be due to the more geographical and climatic similarities with the Eastern Black Sea region. Many researches were supported that fatty acid profile and multivariate data analysis applicable for discrimination geographical origin and detecting adulteration of fruits (Amorello, Orecchio, Pace, & Barreca, 2015; Cortĕs, Garcǐa, Malherio, Guardiola, & Pereira, 2013; Esteki, Farajmand, Kolahderazi, & Gandara, 2017). According to the Rutkowska, Bialek, Adamska, and Zbikowska (2015), fatty acid profiles could serve as markers of geographical origin of cream products. Diraman, Saygi, and Hisil (2010) found that their prediction model was correctly classified by 84.6% of the samples from five different regions. D'Archivio, Giannitto, Incani, and Nisi (2014) reported that they predicted correctly classified more than 80% of the 27 Italian saffron spices using mineral composition.

## 4 CONCLUSION

The compositions of fatty acid in hazelnut samples for Black Sea regions in Turkey were investigated in order to detection of geographical origin by ANOVA and linear discriminant analysis, and significant differences were maintained on the fatty acid compositions (C16:0, C16:1, C18:2, C18:3, C20:0, SFAs, and PUFAs). The results were provided that about 86.2% of correct prediction performed by linear discriminant analysis.

## CONFLICT OF INTEREST

The authors have no conflicts of interest to declare.
